# Simulation of Subrapid Solidification and Secondary Cooling for the Strip Casting of IF Steel

**DOI:** 10.3390/ma14185274

**Published:** 2021-09-13

**Authors:** Wanlin Wang, Song Mao, Hualong Zhang, Cheng Lu, Peisheng Lyu

**Affiliations:** 1 School of Metallurgy and Environment, Central South University, Changsha 410083, China; wanlin.wang@gmail.com (W.W.); 193511003@csu.edu.cn (S.M.); yehuanzhl@163.com (H.Z.); lucheng_0624@126.com (C.L.); 2 National Center for International Research of Clean Metallurgy, Central South University, Changsha 410083, China

**Keywords:** interstitial-free steel, strip casting, subrapid solidification, massive transition, secondary cooling, coiling

## Abstract

A combination of droplet solidification tester and confocal laser scanning microscope was used to simulate subrapid solidification and secondary cooling process pertinent to the strip casting. The IF steel droplet had a delamination structure and the bottom part went through sub-rapid solidification. During secondary cooling, γ/α transformation mechanism belonged to interface-controlled massive transformation and the ferrite grains grew quickly. With the increase of cooling rate, the γ/α transformation temperature decreased and the incubation period and phase transformation duration reduced. The hardness showed a slight increase due to fine-grain strengthening. With coiling temperature increasing from 600 °C to 800 °C, the grain size became larger, precipitates became coarse, and defects in grain were recovered. Consequently, the hardness decreased.

## 1. Introduction

As a near-net-shape casting technology, strip casting has realized the century-old dream of directly producing ultrathin steel strip from molten steel [1,2]. Different from conventional casting and rolling technologies, strip casting process mainly composes of casting thin strip from liquid steel, in-line hot rolling and secondary cooling, following with coiling [3]. Since this revolutionary technique eliminates reheating and repeated hot-rolling steps, it can save a large amount of energy, reduce CO_2_ emission, simplify operating process, and reduce investment costs [4,5]. Because of the absence of molten slag, molten steel can directly contact with the water-cooled copper mold, and thus achieve a cooling rate of 1000–10,000 K/s (subrapid solidification) [1]. This would bring many benefits on the solidification microstructure, such as high solid solubility of alloying elements, refinement of grain size, attenuation of elements segregation, and crystallographic texture [6].

Since H. Bessemer first conceived strip casting in 1846, several strip casting technologies have been researched and developed, such as single- and twin-roll casting and single- and twin-belt casting. Among them, twin-roll casting is the most popular and mature one and has been commercialized in NUCOR Steel. However, NUCOR’s Castrip^®^ process mainly focuses on plain carbon steels [2]. Many research and development (R&D) projects on strip casting have been initiated worldwide since 1980, but there are still many challenges in improving productivity, alleviating surface defects, and producing high alloy steels, etc. Three main types of research methods have been developed: industrial or pilot experiment, numerical simulation, and thermal simulation. However, it is usually costly to conduct industrial or pilot strip casting experiments. Thanks to the progress in computer science, numerical simulation is considered to be an efficient method to simulate heat transfer behavior [7], flow field, and solidification behavior [8] of the conventional continuous casting process. However, when it comes to subrapid solidification process, which is a complicated nonequilibrium process with lacking thermal–physical parameters and boundary conditions, prediction of solidified structure by numerical simulation still has a long way to go.

Consequently, laboratory-scaled thermal simulation equipment was developed for the fundamental studies on strip casting. Interfacial heat transfer between melt and mold has a significant effect on the microstructure and surface quality of steel strip, and the microstructure has a great effect on mechanical properties such as strength and toughness. Zhai [9] developed a flipping pouring apparatus in which molten steel flows into a crucible with a chill copper wall in it. This facility can be used to study heat transfer and solidification of conventional slab cast. However, the cooling rate and flow field are quite different from the strip casting process. Strezov [10] designed an experimental apparatus named the dip tester to approximate the initial contacting conditions between molten steel and copper mold during strip casting by immersing a copper substrate into molten steel. Then, the dip tester was upgraded in Central South University [11,12,13]. A steel strip with a thickness of 0.8–3 mm can be obtained for microstructure and properties examination. Cramb [14] built a droplet solidification tester in which molten steel directly drops onto a chill copper substrate to study factors affecting initial solidification behavior during strip casting, such as surface oxide films [14,15], superheat [16] and the roughness of substrate [17]. Few research studies went further to study related microstructure and pertinent thermal schemes.

Interstitial-free (IF) steel is a steel product in which the interstitial elements C and N are fixed to TiC(N) or NbC(N) by sufficient amount of Ti or Nb [18]. Therefore, IF steel has an excellent deep-drawing performance. With the good combination of low yield strength, high plastic anisotropy ratio, and excellent weldability, IF steel has been widely used in automobile manufacturing industry, household appliances, and so on [19]. Numerous works have been conducted on IF steel to remove interstitial elements [20,21] and improve formability [22]. From industrial viewpoints, IF steel is generally produced by the conventional continuous casting and rolling processes, during which reheating and repeated hot rolling steps escalate operation difficulty and energy consumption greatly. In terms of the apparent advantages of strip casting technique, it is of great significance to carry out fundamental research studies on the solidification process, solid-state phase transformation, and mechanical properties for the strip casting of IF steel. However, related studies have not been reported at present.

It is believed that the solidification and solid phase transformation is quite different when provided with a high cooling rate. At slow cooling rate, undercooled austenite pretends to transform into polygonal ferrite. As the cooling rate increases, massive transformation takes over [23]. Massive transformation is a thermally activated noncooperative phase transformation. Interface control, composition invariance, and irregular boundaries are the main features of massive transformation [24,25,26,27]. Based on in situ observation on ultralow carbon steel, Lee [28] revealed that the acicular Widmanstätten ferrite was also formed through massive transformation mechanism. However, the transformation mechanism, such as dynamics of nucleation and growth, is still unclear.

In this study, the droplet solidification tester was used to obtain the subrapid solidified IF steel sample. Then, a confocal laser scanning microscope was used on the subrapid solidified sample to simulate secondary cooling pertinent to strip casting process, by which in situ observation of high-temperature phase transformation and grain growth was achieved. Finally, the effects of cooling rate and coiling temperature on microstructure evolution and properties of IF steel were discussed.

## 2. Experimental Details

### 2.1. Experimental Apparatus

**Figure 1:**
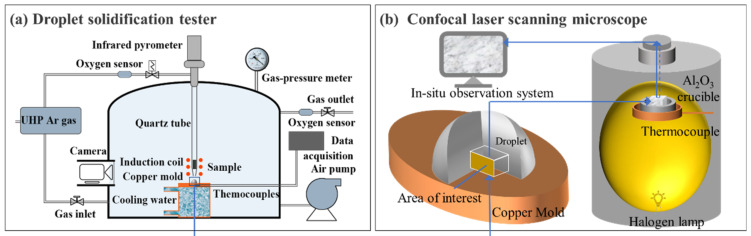
The schematic illustration of (**a**) droplet solidification tester and (**b**) confocal laser scanning microscope.

The droplet solidification tester was designed for simulating the subrapid solidification pertinent to the strip casting by impinging molten steel onto a water-cooled substrate. It was first developed at Carnegie Mellon University [14] and further upgraded at Central South University [16]. The schematic of the droplet solidification tester is shown in Figure 1a, which is mainly comprised of a heating system, ejection system, cooling system, and atmosphere control system. A cylindrical steel specimen is held in a vertical quartz tube with a small hole at bottom. The induction coil outside the quartz tube can heat and melt the specimen in several seconds. The temperature of steel specimen is measured by an infrared pyrometer and transmitted to a proportional–integral–derivative (PID) controller, which can adjust the power of the induction furnace to keep the molten steel at a certain temperature. Under the impulsion of pressurized high-purity argon, the molten steel can be ejected and impinged onto a water-cooled copper mold, and then it starts solidifying. At the same time, the real-time process of droplet ejection and solidification is recorded with a CCD camera. In order to control the atmosphere, the sample and copper mold are placed in a bell jar. With the help of deoxidizer and oxygen sensor, the oxygen partial pressure inside can be adjusted precisely in a wide range.

Confocal laser scanning microscope (CLSM, VL2000DX-SVF18SP) (Yonekura MFG. Co. Ltd., Osaka, Japan) was used to simulate the secondary cooling and coiling processes pertinent to the strip casting of IF steel. CLSM is equipped with an elliptical furnace chamber plated with gold (Figure 1b), in which a halogen lamp was used to heat the sample. The thermocouple with disk shape was used to hold the Al_2_O_3_ crucible and measure the sample temperature. The temperature schedule of the sample can be programmed in advance as we need. A laser radiation is used to form a clear real-time image by scanning the sample surface. When austenite to ferrite transformation takes place, macroscopic volume change causes surface relief; therefore, the solid-state phase transformation can be observed in situ.

### 2.2. Experimental Procedure

**Figure 2:**
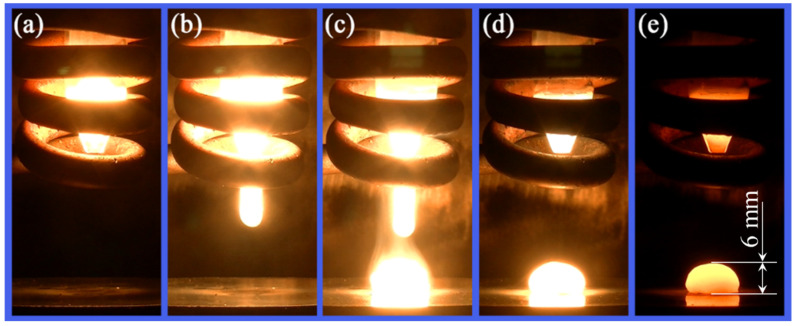
Ejection and solidification process during droplet solidification test: (**a**) melting of the steel sample at target temperature, (**b**,**c**) ejection of liquid steel, (**d**,**e**) solidification on chill copper mold.

The IF steel studied in this work is taken from the slab produced by conventional continuous casting process, whose chemical composition is shown in Table 1. Usually, the average shell cooling rate during conventional continuous casting is about 12 °C/s [3], which is far lower than that of strip casting. First, the IF steel slab was cut into cylinders with a diameter of 6 mm and length of 15 mm, then polished with 400-grit metallographic sandpaper to remove the oxidized layer. Next, the cylinder sample was put into the quartz tube of droplet solidification tester. Then, the sample was heated and melted by the induction coil (Figure 2a). As soon as it reaches the target temperature (1586 ± 3 °C), the liquid sample was ejected onto the copper mold (Figure 2b,c), followed by rapid solidification to form a hemisphere droplet with a radius of 6 mm (Figure 2d,e). Since the bottom of the droplet made direct contact with the copper substrate, it should have gone through subrapid solidification and is of greater interest. The following tests focus on the region that is 0.5–1.5 mm away from the bottom surface of droplets.

**Figure 3:**
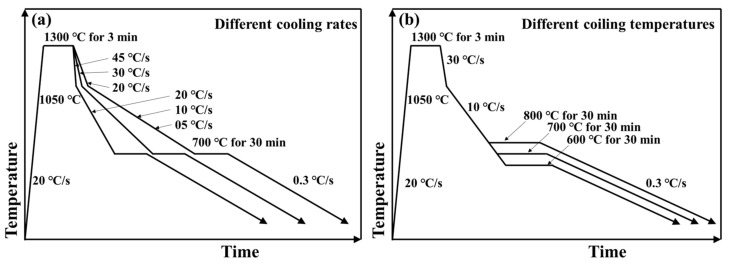
Temperature history in CLSM: (**a**) different cooling rates, (**b**) different coiling temperatures.

The second-stage experiment aims at simulating secondary cooling and coiling after subrapid solidification of IF steel. A 5 × 5 × 3 mm^3^ subrapid solidified sample was cut from the bottom of the solidified droplet, as is shown in Figure 1b. The temperature history of the sample in the CLSM is illustrated in Figure 3. In order to simulate the grain size and distribution of prior austenite characterized by the as-cast strip, the samples were heated to 1300 °C at a rate of 20 °C/s, and then held for 3 min [29], as shown in Figure 3. Similar schedule for simulating prior austenite grain size was also applied in Pereloma’s work [30]. After austenization, the sample was cooled to 600–800 °C with different cooling rates and then held for 30 min to simulate the coiling process. For the cooling process from 1300 °C to 1050 °C, three different cooling rates were set, i.e., 45 °C/s, 30 °C/s, and 20 °C/s. When the temperature was below 1050 °C, the cooling rates were set as 20 °C/s, 10 °C/s, and 5 °C/s, respectively. Consequently, there were three sets of different cooling rates in this study, denoted by 45–20 °C/s, 30–10 °C/s, and 20–05 °C/s, respectively. The coiling temperatures were 600 °C, 700 °C and 800 °C, respectively. Figure 3a shows the first series of experiments that cooling at different rates following with coiling at 700 °C. Figure 3b shows another series of experiments that coiling at different temperatures after cooling with the same cooling schedule of 30–10 °C/s.

### 2.3. Analytical Methods

The solidified droplet prepared by droplet solidification tester was cut into halves along the longitudinal direction. The initial IF slab and droplet samples were metallographically prepared through the standard procedure, followed by picric acid or nitric acid etching for the microstructure examination through optical microscopy (OM, MR5000) (Jiangnan Yongxin Optical Co., LTD, Nanjing, China). Factsage 7.2 was used to figure out equilibrium phase transformation temperature and precipitation thermodynamics. The solid-state phase transformation was in situ observed by CLSM. After the simulation of secondary cooling and coiling, the samples were also metallographically prepared, and then etched by nitric acid for OM examination. Hardness was measured under the load of 2 kg with 10 s retention using a digital Vickers hardness tester (DVHT, HVS-5) (Laizhou Huayin Test Instrument Co. LTD, Yantai, China). Six individual points were tested for each sample at the same region (0.2-0.3 mm away from the bottom surface of droplet). The average and deviation were calculated. Yield strength (*YS*) and ultimate tensile strength (*UTS*) were estimated using the following formulae [31]:

(1) $$YS=2.87\times H_{V}-90.7$$

(2) $$UTS=3.734\times H_{V}-99.8$$

## 3. Results and Discussion

### 3.1. Thermodynamics Calculation and Microstructures of IF Steel Samples

**Figure 4:**
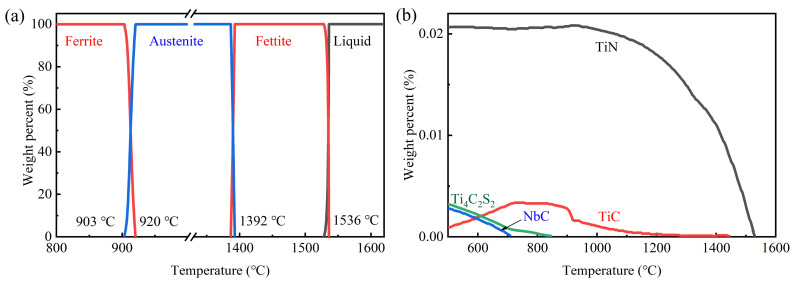
Thermodynamics calculation: (**a**) phase evolution and (**b**) precipitation as a function of temperature.

Figure 4 shows the equilibrium phase diagram calculated by Factsage. Owing to the low alloying element content, the phase transformation temperatures of liquid (L)→ ferrite (δ), ferrite → austenite (γ), and austenite → ferrite (α) are 1536 °C, 1392 °C, and 920 °C, respectively (Figure 4a), which are close to those of pure iron. According to the thermodynamics calculation (Figure 4b), the precipitate sequence can be speculated to be TiN, TiC, NbC, and Ti_4_C_2_S_2_ as the temperature decreases. TiN and TiC form at a high temperature and consume the vast majority of Ti. At 700 °C, part of C is substituted by S, so that TiC transforms into Ti_4_C_2_S_2_. In addition, redundant C diffuses out and combines with Nb to form NbC around Ti_4_C_2_S_2_. Similar phenomena were also reported by Hua et al. [32].

**Figure 5:**
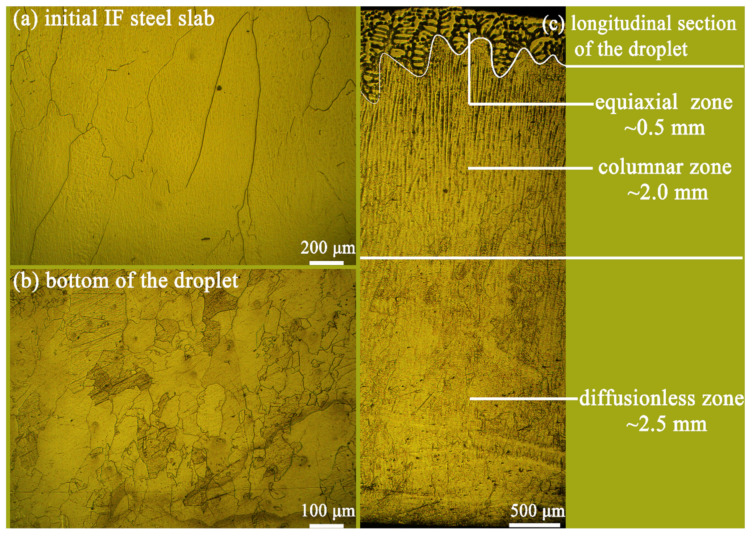
Microstructures of IF steel samples: (**a**) initial IF steel slab, (**b**) bottom, and (**c**) longitudinal section of the solidified droplet. (**a**,**b**) were etched by nitric acid and (**c**) were etched by picric acid.

Figure 5 shows the optical microstructures of different IF steel samples. The steel materials studied in this work (named initial IF slab hereafter) were produced by a conventional continuous casting process. Figure 5a shows its coarse columnar ferrite grains, whose diameter is 300–400 μm and length is longer than 1000 μm. After the droplet test, however, the bottom part of the droplet shows a refined ferrite grain of only dozens of microns (Figure 5b). The heat transfer between droplet and substrate can be considered to be nearly one dimension, which is perpendicular to the substrate surface. The solidification structure is significantly influenced by the heat transfer between steel droplet and copper substrate. As is shown in Figure 5c, almost all dendrites are parallel to the heat transfer direction. As a result, three distinct microstructures were formed within a single droplet, i.e., diffusionless zone at the bottom, columnar zone in the center, and equiaxial zone at the top.

In the bottom part of droplet, the molten steel made contact with water-cooled copper substrate directly. Therefore, it solidified so rapidly that solute atoms were frozen in Fe matrix, forming the diffusionless zone. The diffusionless zone measures up to 2.5 mm in thickness. Dendrite structures can hardly be observed after etching with picric acid in this zone. The grains are quite fine, irregular, and elongated along the direction of heat flow.

In the center part of droplet, dendrite structure perpendicular to the surface of the copper substrate is observed. This 2-mm-thick section is called the columnar zone. Grains in this part are much coarser than that at the bottom. It is reported that primary dendrite arm spacing (PDAS) is governed by the cooling rate and carbon content in steel. H. Jacobi established the following formula to estimate PDAS [33]:

(3) $$\lambda 1=\frac{283}{C_{R}{}^{0.49}}$$

where λ1 represents primary dendrite arm spacing measured as 10–15 μm and *C_R_* is the estimated cooling rate. The cooling rate is estimated to be 400–1000 °C/s in the center part of the solidified droplet. The cooling rate of the bottom part would be larger because it was closer to the chill copper substrate. Therefore, it can be concluded that the bottom part of droplet went through a subrapid solidification (>1000 °C/s).

Equiaxial grains with coarse primary and secondary dendrite were formed near the top of solidified droplet. This is attributed to the slowly cooling rate and multidirectional heat transfer of the top part of solidified droplet.

### 3.2. Austenite Transformation and Growth during Heating and Isothermal Processes

**Figure 6:**
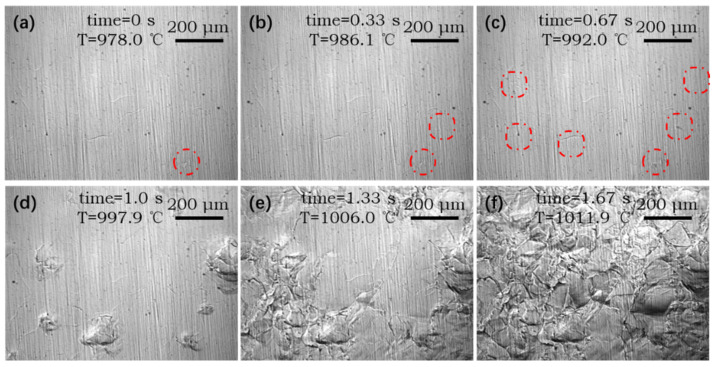
In situ observation of austenite transformation from ferrite during the heating process of subrapid solidified sample at a heating rate of 20 °C/s: (**a**–**f**) are snapshots at 0s, 0.33 s, 0.67 s, 1.0 s, 1.33 s, and 1.67 s from 978 °C, respectively. Visible nucleation sites are denoted in red circles.

As shown in Figure 3, a series of CLSM experiments were conducted on subrapid solidified samples. Typical CLSM pictures of austenite transformation during continuous heating process are shown in Figure 6. The beginning temperature of transformation from ferrite to austenite during the heating process of subrapid solidified sample is 978 °C (Figure 6a), which is much higher than that for the case of equilibrium phase transformation (903 °C, Figure 4a). The number of nuclei increases during the continuous heating process. Nucleation rate is determined by the atomic mobility through the γ/α interface, which is given by an Arrhenius term [34]:

(4) $$\dot{N}\left(T\left(t\right)\right)=N_{0}exp\left(\frac{-Q_{n}}{RT\left(t\right)}\right)$$

where $\dot{N}\left(T\left(t\right)\right)$ is nucleation rate per unit volume, *N_0_* is temperature-independent constant, *Q_n_* is activation energy for atom migration through the γ/α interface, *R* is the universal gas constant (=8.31 J/mol.K), and *T* is the absolute temperature(K). This formula indicates an exponential increase of nucleation rate with the increase of temperature. During the formation of austenite, new austenite phases grew into old ferrite grains rapidly and impinged with each other (Figure 6d–f). About 1.67 s later, the morphology of sample surface tended to be stable while the temperature reached 1112 °C (Figure 6f), indicating the accomplishment of austenite transformation. It can be found that the austenite grain size is ~80 μm when the austenization finished.

**Figure 7:**
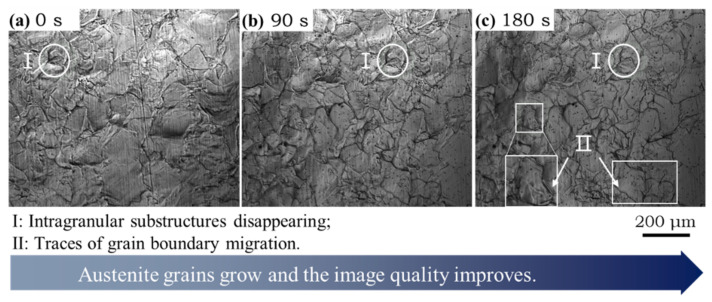
Austenite growth of subrapid solidified sample held at 1300 °C:(**a**–**c**) are snapshots at 0 s, 90 s, and 180 s from 1300 °C, respectively.

Typical CLSM pictures of austenite growth during isothermal process are shown in Figure 7. Compared with the heating process, there were some obvious features during isothermal process at 1300 °C. First, some surface relief within grains disappeared and the grain boundaries became clearer, as indicated by region I. The migration of grain boundaries can be identified in Figure 7c (region II). This is direct evidence of grain growth by boundary migration during isothermal process. After being held at 1300 °C for 180 s, the average austenite grain size of subrapid solidified sample reached ~120 μm, which is close to 124 µm of Nb microalloying steel [35] produced by the CASTRIP^®^ (Castrip^®^ LLC, Charlotte, NC, USA) process and 117 ± 44 μm of dual phase steel [29] produced by the dip tester.

### 3.3. Ferrite Transformation of Subrapid Solidified IF Steel at Different Cooling Rates

**Figure 8:**
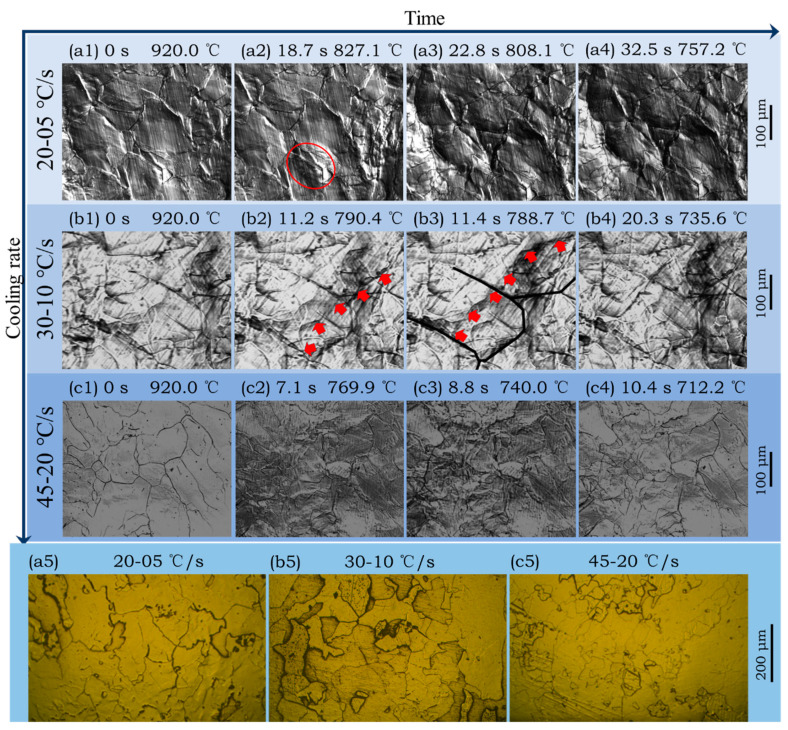
(**a1**–**c4**): Ferrite transformation of subrapid solidified samples at three different cooling rates and (**a5**–**c5**): corresponding optical microstructure etched by nitric acid. Circle in (**a2**) delineates ferrite nucleation at prior austenite boundaries. Red arrows in (**b2**,**b3**) depict a route of γ/α interface migration. Black curves in (**b2**,**b3**) outline prior γ boundaries.

**Figure 9:**
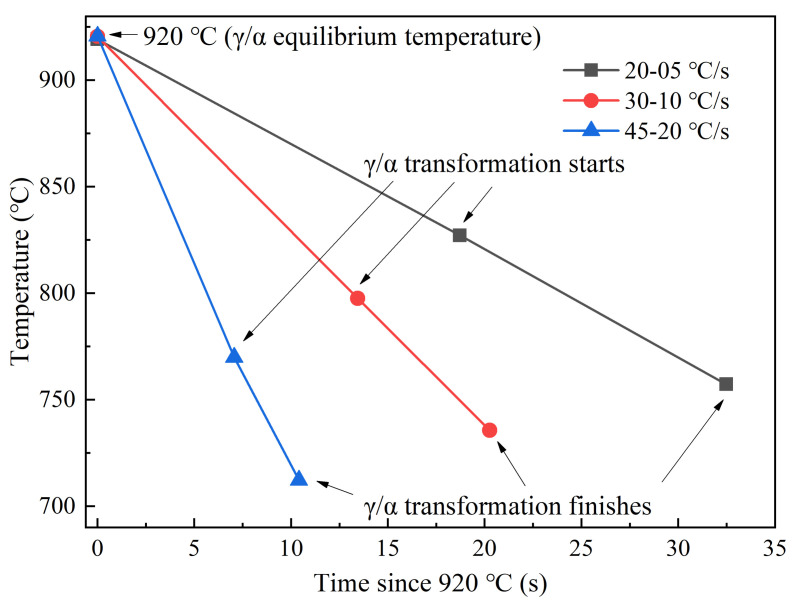
Temperature and duration of phase transformation from austenite to ferrite at different cooling rates. Time = 0 s corresponds to initial transformation temperature from austenite to ferrite at equilibrium condition.

Temperature history used in this section is shown in Figure 3a. According to CLSM videos, ferrite transformation occurred successively at different cooling schedules during the cooling process from 1300 °C to coiling temperature of 700 °C. Some key frames are listed in Figure 8. 920 °C is the equilibrium transformation temperature calculated by Factsage. For the cooling schedule of 20–05 °C/s, ferrite phase transformation starts at 830.6 °C. Ferrite mainly nucleates at the austenite grain boundaries, where energy fluctuation and structural fluctuation are more likely to meet the transformation condition, as in shown in Figure 8(a2). At about 808.1 °C, the surface calms down and does not change until 757.2 °C (Figure 8(a3,a4)). Similar processes of phase transformation can also be observed for cooling schedules of 30–10 °C/s (Figure 8(b1–b4)) and 45–20 °C/s (Figure 8(c1–c4)), but temperature and duration of ferrite transformation are different for different cooling schedules. As is abstracted in Figure 9, with the increase of cooling rate from 20–05 °C/s to 30–10 °C/s and 45–20 °C/s, the γ/α transformation temperature decreases from 830 °C to 801 °C and 783 °C, while the incubation period and phase transition duration reduces. This is because the high cooling rate can provide a remarkable supercooling for the transformation from austenite to ferrite, and thus promotes ferrite nucleation and speeds up the growth process. In addition, it can also be found that the microstructures get refined with the increase of secondary cooling rate. This is because a higher cooling rate can increase the nucleation rate of ferrite and refine the final microstructure.

Due to the ultralow content of carbon, long-range diffusion is not necessary at γ/α interface during the ferrite transformation. The phase transformation belongs to interface-controlled type. Thermally activated atoms cross the γ/α interface and rearrange as ferrite quickly. The arrows in Figure 8(b2,b3) indicate the position and growth direction of α grains. The growth rate was about 350 mm/s on average. Some α grains grew across prior γ grains, indicating that the γ/α interface was incoherent. Figure 8(a5–c5) show the optical microstructures of the above three samples after coiling at 700 °C. They all have ragged ferrite grains with irregular boundaries. All of these features match well with massive transformation [28,36].

### 3.4. Effect of Coiling Temperature on the Microstructure of Subrapid Solidified IF Steel 

**Figure 10:**
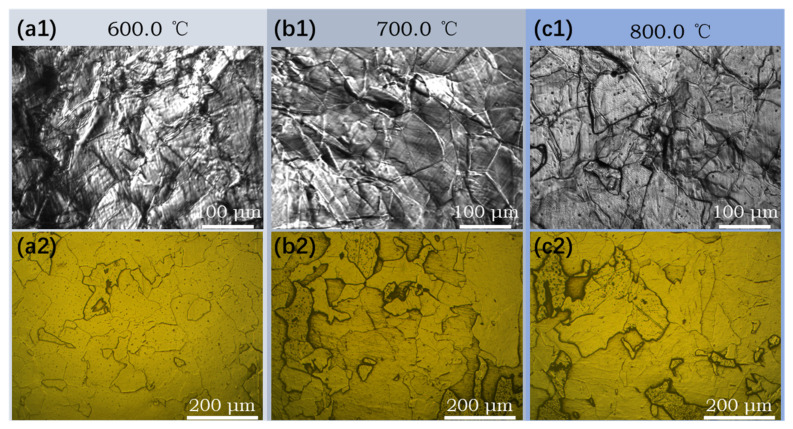
CLSM pictures and optical microstructures after coiling simulation at (**a**) 600 °C, (**b**) 700 °C, and (**c**) 800 °C, respectively.

**Figure 11:**
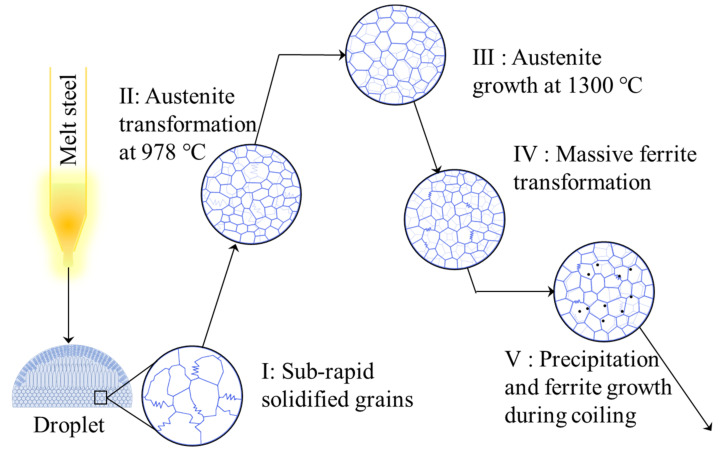
Schematic diagram of the microstructure evolution during the simulated strip casting process of IF steel.

Temperature history used in this section is shown in Figure 3b, by which subrapid solidified samples with different coiling temperatures can be obtained. Figure 10 shows the CLSM pictures and optical microstructures of samples after coiling simulation at 600 °C, 700 °C, and 800 °C, respectively. The microstructures are identified to be ferrite with different grain sizes and irregular boundaries. When coiled at 600 °C and 700 °C, the average grain sizes of the samples are quite similar. According to Figure 9, these coiling temperatures are lower than the finishing temperature of ferrite transformation (771 °C) at the cooling schedule of 30–10 °C/s, that is, the coiling process was conducted after ferrite transformation. Due to the relative low temperature and pinning effect of precipitate, the ferrite grains did not grow obviously during the coiling process. As is shown in Figure 9, the start temperature of ferrite transformation is 801 °C at the cooling schedule of 30–10 °C/s, so the sample went through an isothermal phase transformation when coiled at 800 °C. Due to a higher γ/α transformation and lower supercooling, the resulting grain size is obviously larger than that of 600 °C and 700 °C coiling conditions. A higher coiling temperature would eliminate several lattice defects generated during cooling. The scavenging effect of titanium and niobium in IF steel not only renders the ferrite matrix nearly interstitial free but also leads to the formation of precipitates. The formation of precipitates is influenced by coiling temperatures during strip casting. As is indicated in Figure 4b, TiN is the dominating precipitate formed during and after coiling. Coiling at a higher temperature would promote the formation and coarsening of TiN and TiC. In contrast, coiling at a lower temperature may result in the insufficient scavenging of nitrogen in solid solution. The formation of Ti_4_C_2_S_2_ and NbC at 600 °C would also enhance the strength of the coiled sample. The whole microstructure evolution process during subrapid solidification, reaustenitization, secondary cooling, and grains growth after coiling is schematically described in Figure 11.

### 3.5. Relationship between Mechanical Properties and Microstructure

**Figure 12:**
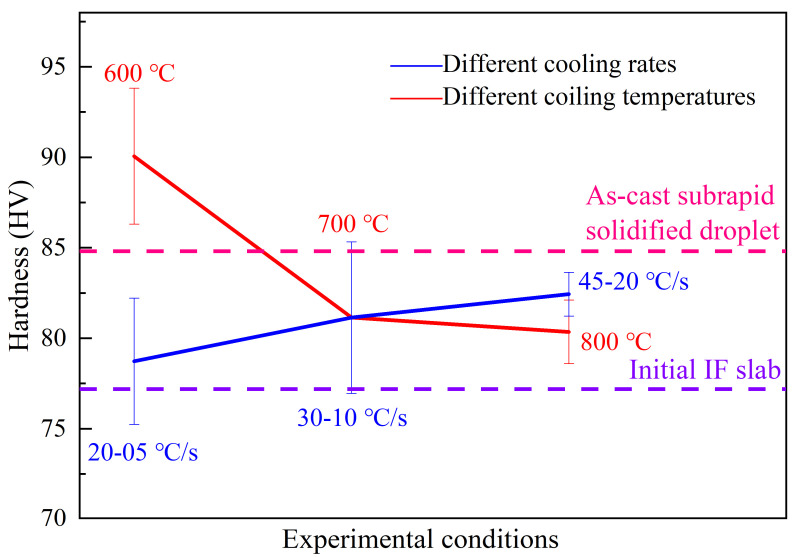
Vickers hardness under different conditions. Error bar represents the standard deviation.

Vickers hardness of samples under different conditions is measured using a Vickers hardness tester. The yield strength and ultimate tensile strength are estimated by the empirical formulae. They show a similar pattern with hardness variation (Table 2). It is well known that there are four basic types of strengthening mechanisms for metals, i.e., solution strengthening, dislocation strengthening, precipitate strengthening, and fine-grain strengthening [37]. As shown in Figure 12 and Table 2, the initial IF slab sample shows the lowest hardness and strength compared with the other samples. This can be attributed to the coarse columnar ferrite grain and low dislocation density due to slow solidification. The hardness or strength of the subrapid solidified sample in as-cast condition is higher than that of the initial IF slab. This is because the subrapid solidified sample has a more refined ferrite structure (Figure 5) and higher dislocation density. A higher dislocation density of the subrapid solidified sample than that of the initial IF slab is caused by the high cooling rate during droplet solidification test. Then, the subrapid solidified sample in as-cast condition was used to simulate the secondary cooling and coiling processes by CLSM. It can be found that the hardness or strength increase with the increase of secondary cooling rate, as shown in Table 2. This can be explained as a higher secondary cooling rate results in a finer ferrite structure (Figure 8(a5–c5). When increasing the coiling temperature, however, the hardness or strength decreases. This is attributed to the coarsening of precipitates, decreasing dislocation density, and increasing ferrite grain size. Furthermore, the hardness or strength of samples coiled at 700 °C or 800 °C are lower than that of as-cast subrapid solidified sample, but the sample coiled at 600 °C presents a highest hardness or strength among all samples. As is shown in Figure 4b, holding at 600 °C can maximize weight percent of precipitations. What’s more, a low temperature can delay the aggregation of precipitation particles. Consequently, coiling at 600 °C can obtain a higher hardness and strength.

## 4. Conclusions

Based on the droplet solidification technique, the CLSM experiment on subrapid solidified droplet was conducted for the first time to simulate secondary cooling and coiling processes pertinent to strip casting, by which in situ observation of high-temperature transformation and grain growth was achieved. Then, the effects of cooling rate and coiling temperature on the microstructure and mechanical properties of IF steel were studied. The main results can be summarized as follows:

(1) The droplet has a hierarchical structure: fine diffusionless zone at the bottom (~2.5 mm), columnar zone in the center (~2.0 mm), and equiaxial zone at the top (~0.5 mm). By measuring dendrite arm spacing in columnar zone, the solidification rate at the bottom is indirectly estimated to be higher than 1000 °C/s, confirming that the droplet tester can simulate subrapid solidification successfully. Fine grains with irregular boundaries were formed in the bottom part of droplet during subrapid solidification. Its hardness is 84.8 HV, higher than that of the initial IF slab (77.7 HV).
(2) After reheating to 1300 °C at the rate of 20 °C/s and held for 3 min, the average austenite grain size of subrapid solidified sample reached ~120 μm. Upon this microstructure, secondary cooling and hot coiling is simulated. With the help of CLSM, interface-controlled massive transformation is directly observed. Ferrite mainly nucleates at the austenite grain boundaries, and the ferrite grains grow quickly (~350 μm/s).
(3) With the increase of secondary cooling rate, the γ/α transformation temperature decreases, and the incubation period and phase transformation duration are reduced. As a result, the hardness shows a slight increase due to fine-grain strengthening.
(4) With coiling temperature increasing from 600 °C to 800 °C, the grain size becomes larger, precipitates such as TiN and TiC become coarser, and lattice defects of grain decrease. Consequently, the hardness of solidified sample decreases.

## Figures and Tables

**Table 1:** Chemical composition of the studied IF steel (Weight percent).

C	N	Si	Mn	P	S	Al	Ti	Nb
0.0008	0.0050	0.005	0.107	0.0113	0.0041	0.0335	0.0189	0.0112

**Table 2:** Vickers hardness and estimated strength.

Samples	Vickers Hardness	Estimated Yield Strength (MPa)	Estimated Ultimate Tensile Strength (MPa)
Initial IF slab	77.7 ± 3.4	132.8	190.4
As-cast subrapid solidified sample	84.8 ± 3.2	153.1	216.8
Cooling-20–05 °C/s	78.7 ± 3.5	135.7	194.2
Coiling-600 °C	90.1 ± 3.8	168.3	236.5
Cooling-30–10 °C/s (Coiling-700 °C)	81.1 ± 4.2	142.7	203.2
Coiling-800 °C	80.4 ± 1.8	140.4	200.3
Cooling-45–20 °C/s	82.4 ± 1.2	146.4	208.0

## Data Availability

The data presented in this study are available on request from the corresponding author.
